# Seaweed-Derived Polysaccharides Attenuate Heat Stress-Induced Splenic Oxidative Stress and Inflammatory Response via Regulating Nrf2 and NF-κB Signaling Pathways

**DOI:** 10.3390/md20060358

**Published:** 2022-05-27

**Authors:** Wen-Chao Liu, Di-Pai Zhuang, Yue Zhao, Balamuralikrishnan Balasubramanian, Zhi-Hui Zhao

**Affiliations:** 1 Department of Animal Science, College of Coastal Agricultural Sciences, Guangdong Ocean University, Zhanjiang 524088, China; liuwc@gdou.edu.cn (W.-C.L.); zhuangdipai@stu.gdou.edu.cn (D.-P.Z.); zhaoyue2@stu.gdou.edu.cn (Y.Z.); 2 Department of Food Science and Biotechnology, College of Life Science, Sejong University, Seoul 05006, Korea

**Keywords:** *Enteromorpha prolifera*, chickens, oxidative stress, inflammatory response, polysaccharides, Nrf2, NF-κB pathways

## Abstract

With global warming, heat stress (HS) has become a worldwide concern in both humans and animals. The ameliorative effect of seaweed (*Enteromorpha prolifera*) derived polysaccharides (SDP) on HS-induced oxidative stress and the inflammatory response of an immune organ (spleen) was evaluated using an animal model (*Gallus gallus domesticus*). In total, 144 animals were used in this 4-week trial and randomly assigned to the following three groups: thermoneutral zone, HS, and HS group supplemented with 1000 mg/kg SDP. Dietary SDP improved the antioxidant capacity and reduced the malondialdehyde (MDA) of the spleen when exposed to HS, regulated via enhancing nuclear factor erythroid 2-related factor-2 (Nrf2) signaling. Furthermore, the inclusion of SDP reduced the levels of pro-inflammatory cytokines and alleviated HS-induced splenic inflammatory response by suppressing the nuclear factor-kappa B (NF-κB) p65 signaling. These findings suggest that the SDP from *E. prolifera* can be used as a functional food and/or feed supplement to attenuate HS-induced oxidative stress and inflammatory responses of the immune organs. Moreover, the results could contribute to the development of high-value marine products from seaweed for potential use in humans and animals, owing to their antioxidant and anti-inflammatory effects.

## 1. Introduction

Heat stress (HS) is emerging as an environmental hazard with global warming, impairing organ health in both humans and animals [1,2]. An average of 1500 HS-related deaths have been reported annually due to the heatwave in the United States [3]. Due to the presence of feathers, high metabolic rate, and a lack of sweating, the broiler chicken (*Gallus gallus domesticus*) is highly sensitive to HS; therefore, it is an ideal animal model to research HS injuries [4]. HS results in physiological dysfunction, including electrolyte imbalance, endocrine disorders, and metabolic abnormalities in chickens [5,6]. Along with multiple physiological disturbances, HS induces the generation of excessive reactive oxygen species (ROS), which negatively affects the redox and immune balance and evokes oxidative stress and inflammation in immune organs, ultimately having a profound impact on immune function and health [3,7,8]. Previous studies have demonstrated that HS results in alterations in the expression of inflammatory cytokines in immune organs [9]. The existing literature reports that HS is associated with nuclear factor erythroid 2-related factor 2 (Nrf2)-mediated oxidative stress and nuclear factor-kappa B p65 (NF-κB p65)-mediated inflammatory response [10,11]. The spleen is one of the key immune organs and plays a vital role in providing immunity [10]. Hence, studies have focused on determining the impacts of HS on the spleen and exploring novel mitigation strategies, as these could be beneficial in extenuating the HS-induced deleterious consequences on immune organs.

Currently, the use of natural polysaccharides as functional supplements and/or therapeutic drugs has attracted great interest due to their multiple biological activities [12]. In this regard, seaweed-derived polysaccharides (SDP), have been shown to alleviate oxidative stress and improve immune response in the immune organs [13,14]. The polysaccharides are the important functional phytochemicals in seaweed *Enteromorpha prolifera* (EP) [15]. EP is widely found across oceans and has been traditionally used as functional food and medicine worldwide, especially in East Asia [16]. Previous studies have confirmed that SDP from EP exhibit various medicinal properties, including antioxidant, antibacterial, and anti-inflammatory effects [17,18,19]. In practice, the recent literature shows that SDP extracted from EP improves the redox status and immune function of model animals [20,21]. Moreover, dietary SDP has been reported to enhance the antioxidant performance and intestinal barrier function in a chicken model [22,23]. Furthermore, supplementing the diet with SDP mitigates oxidative stress and apoptosis in immune organs resulting from exposure to toxins; the Nrf2/heme oxygenase-1 (HO-1) signaling pathway is activated by dietary SDP, thus upregulating the expression of downstream antioxidant molecules [24]. However, studies reporting the effects of SDP on the amelioration of HS-induced oxidative stress and inflammatory response of the spleen in broiler chicken as an animal model are limited. Therefore, the current study aimed to evaluate the protective effects of dietary SDP on HS-induced splenic oxidative stress and the immune response in a chicken model and elucidate the underlying molecular mechanisms. The present research can contribute to increase the use of high-value products from seaweed in animals and humans.

## 2. Results

### 2.1. Composition Characterization of SDP

**Figure 1:**
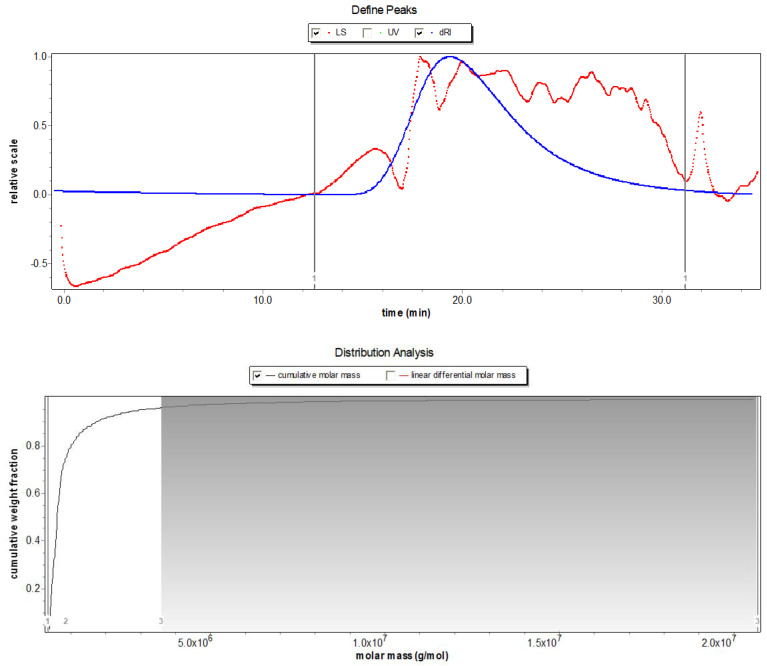
Molecular weight chromatogram of polysaccharides derived from the seaweed *Enteromorpha prolifera* using gel permeation chromatography (GPC). LS, light scattering; UV, ultraviolet spectroscopy, dRI, differential refraction.

**Figure 2:**
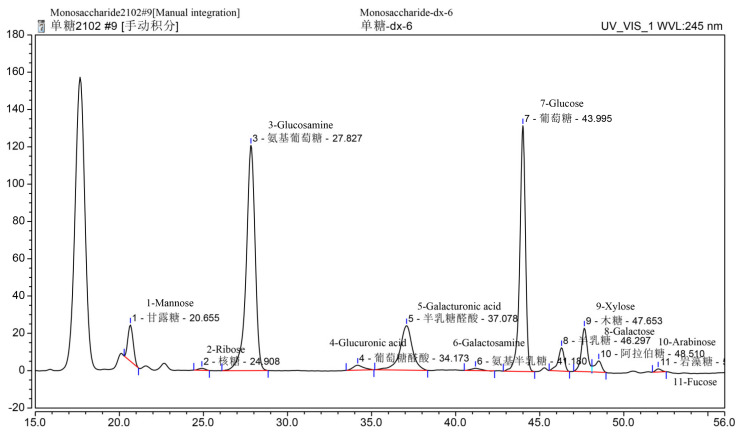
Monosaccharide composition chromatogram of polysaccharides derived from the seaweed *Enteromorpha prolifera* using high-performance liquid chromatography (HPLC). Above each Chinese word is the corresponding English translation.

Based on the phenol sulfuric acid method, Bradford assay, barium sulfate-gelatin turbidimetric analysis, and m-hydroxybiphenyl colorimetric assay, the total polysaccharide, sulfate, uronic acid, moisture, and protein content in SDP were determined to be 53.32%, 19.87%, 12.66%, 3.58%, and 1.65%, respectively; and the unspecified compounds were 8.92% (Table 1). Using gel permeation chromatography (GPC) (Figure 1), the molecular weight findings of SDP were as follows: number-average molecular weight (Mn) = 640.3 kDa, weight-average molecular weight (Mw) = 1156 kDa, peak-average molecular weight (Mp) = 39.11 kDa, and Z-average molecular weight (Mz) = 17630 kDa, and Mw/Mn = 1.805. The monosaccharide composition of SDP is presented in Table 2 and Figure 2. SDP was found to be mainly composed of 50.81% glucosamine (GlcN), 27.70% glucose (Glc), 11.75% galacturonic acid (GalA), 2.78% mannose (Man), 2.63% xylose (Xyl), 1.95% galactose (Gal), 0.93% arabinose (Ara), 0.76% glucuronic acid (GlcA), 0.27% fucose (Fuc), 0.25% galactosamine (GalN), and 0.17% ribose (Rib).

**Figure 3:**
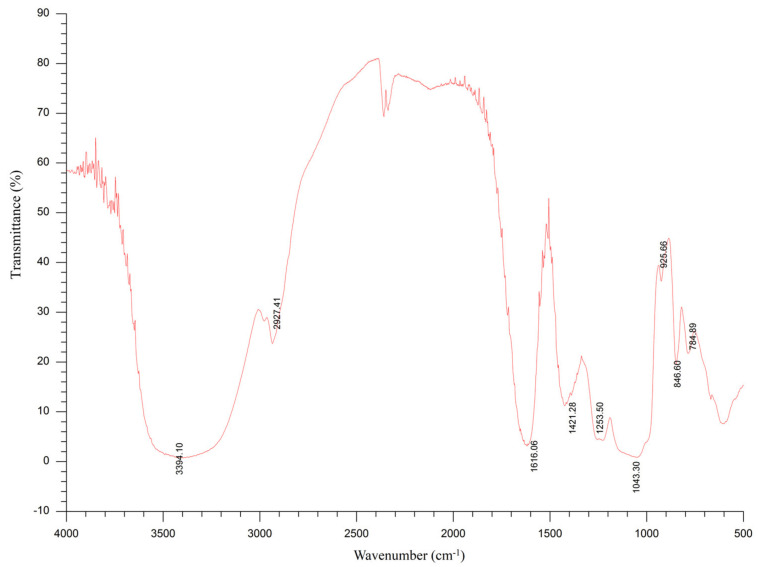
Fourier-transform infrared (FT-IR) spectroscopy of polysaccharides derived from the seaweed *Enteromorpha prolifera*.

The FT-IR spectra of SDP are shown in Figure 3. The absorption trough of wave at 3600–3200 cm^−1^ (the trough point is 3394 cm^−1^) is the characteristic of sugars, and the trough in this range represents -OH stretching vibrations. Furthermore, the absorption trough at 2927 cm^−1^ is assigned to C-H stretching vibrations. The absorption trough at 1616 cm^−1^ is attributed to the crystal water. An absorption trough can be seen at 1421 cm^−1^, which is assigned to the stretching vibration of C-O. The absorption troughs at 1253 cm^−1^ and 1043 cm^−1^ are the variable angle vibration of -OH. The absorption trough at 925 cm^−1^ represents the pyran ring stretching vibration. The absorption trough at 846 cm^−1^ is attributed to the C-H variable angle vibration of the α-terminal epimerization of the pyran ring.

### 2.2. Histology, Apoptosis Analysis and Relative Weight of Spleen

**Figure 4:**
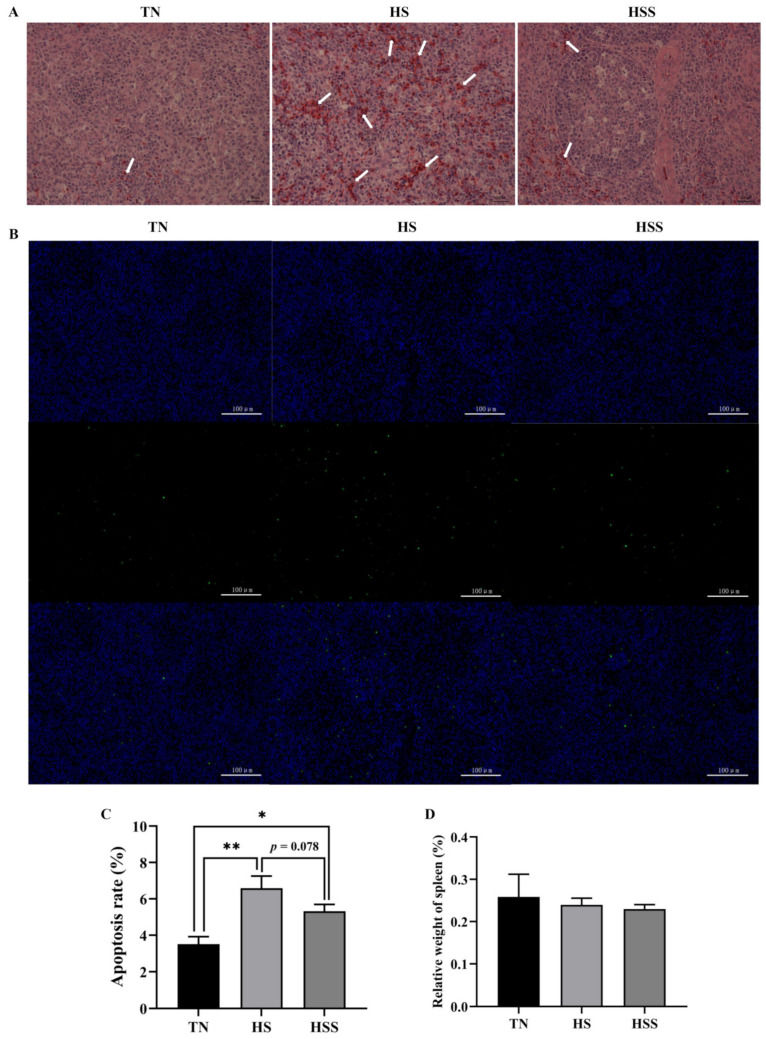
Histology (**A**), cell apoptosis (**B**,**C**), and relative weight of spleens (**D**) subjected to heat stress and after treatment with dietary seaweed-derived polysaccharides. * *p* < 0.05, ** *p* < 0.01, no superscript marks indicated that *p* > 0.10. TN, thermoneutral zone; HS, heat stress; HSS, HS group supplemented with 1000 mg/kg seaweed-derived polysaccharides. Scale bars of histology (**A**), 400× are 25 μm and scale bars of cell apoptosis (**B**), 200× are 100 μm. Arrows (**A**), indicated that the infiltration of inflammatory cells, cell death, and destruction of the cell structure.

As illustrated in Figure 4A, no histopathological changes were seen in the thermoneutral zone (TN) group. However, there was infiltration of inflammatory cells, cell death, and destruction of the cell structure in the spleen from the HS group. Dietary SDP supplementation alleviated inflammatory cell infiltration and reduced the destruction of cell structure. Cell apoptosis results revealed an increase in splenic apoptosis rate after HS exposure (Figure 4B,C, *p* < 0.05), but dietary SDP supplementation did not protect against HS-induced apoptosis of the spleen (*p* > 0.05). Additionally, there were no significant differences in the relative weight of the spleen between groups (Figure 4D, *p* > 0.05).

### 2.3. Antioxidant Capacity

**Figure 5:**
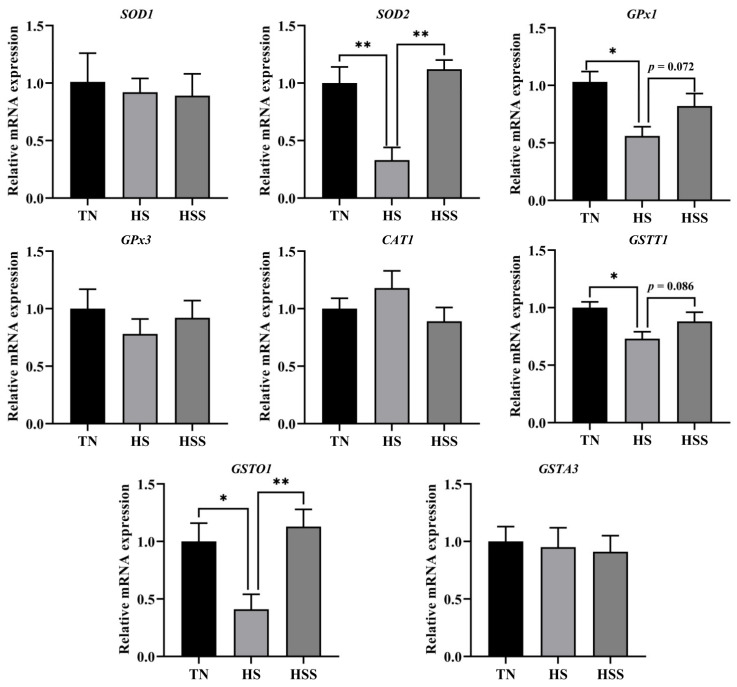
Splenic relative mRNA expression of antioxidant enzyme-related genes after being subjected to heat stress and treatment with dietary seaweed-derived polysaccharides. * *p* < 0.05, ** *p* < 0.01, no superscript marks indicated that *p* > 0.10. TN, thermoneutral zone; HS, heat stress; HSS, HS group supplemented with 1000 mg/kg seaweed-derived polysaccharides. The abbreviations for the detected parameters (SOD, CAT, GPx, GSTT, GSTA and GSTO) are superoxide dismutase; catalase; glutathione peroxidase; glutathione S-transferase theta; glutathione S-transferase alpha and glutathione S-transferase omega, respectively.

Antioxidant-related biochemical parameters of the spleen are presented in Table 3. Compared with those in the TN group, the total antioxidant capacity (T-AOC), total superoxide dismutase (T-SOD), and glutathione-S transferase (GST) activities were reduced, but the malondialdehyde (MDA) and ROS levels in the spleen were elevated by HS (*p* < 0.05). However, SDP supplementation improved T-AOC and GST activities and reduced MDA concentration in the spleen (*p* < 0.05). As depicted in Figure 5, the relative mRNA expression of *SOD2,* glutathione peroxidase *(GPx) 1, GSTT1,* and *GSTO1* in the spleen were downregulated after HS exposure (*p* < 0.05). However, the relative mRNA expression of *SOD2* and *GSTO1* in the spleen from the HS group was upregulated after supplementation with dietary SDP (*p* < 0.01).

### 2.4. Cytokine Levels and Cytokine Gene Expression

**Figure 6:**
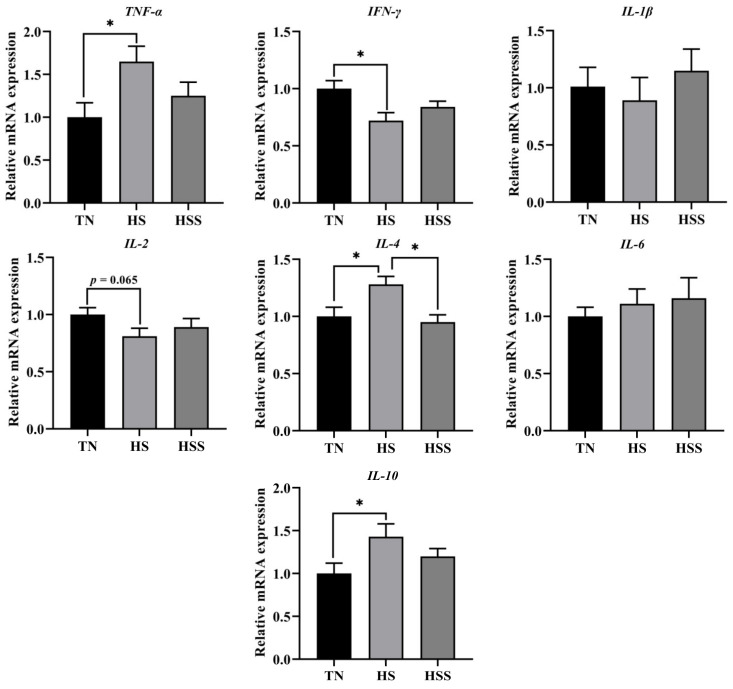
Relative mRNA expression of splenic cytokine genes after subjecting to heat stress and treatment with dietary seaweed-derived polysaccharides. * *p* < 0.05, no superscript marks indicated that *p* > 0.10. Abbreviations for the detected parameters (TNF-α, IFN-γ and IL) are tumor necrosis factor-α, interferon-γ and interleukin, respectively.

The cytokine levels of the spleen are shown in Table 4. The HS group had higher splenic tumor necrosis factors (TNF)-α, interleukin (IL)-4, and IL-10 concentrations, and lower splenic interferon (INF)-γ and IL-2 concentrations compared with the TN group (*p* < 0.05). In contrast, SDP supplementation decreased TNF-α, IL-4, and IL-10 levels and increased IL-2 levels in the spleen from the HS group (*p* < 0.05). As indicated in Figure 6, the HS group exhibited lower *IFN-γ* mRNA expression (*p* < 0.05). HS exposure increased *TNF-α, IL-4,* and *IL-10* mRNA expression in the spleen compared with those in the TN group (*p* < 0.05). In contrast, dietary supplementation with SDP reduced splenic *IL-4* mRNA expression (*p* < 0.05).

### 2.5. Expression of Nrf2 and NF-κB p65 Signaling Pathway–Related Molecules

**Figure 7:**
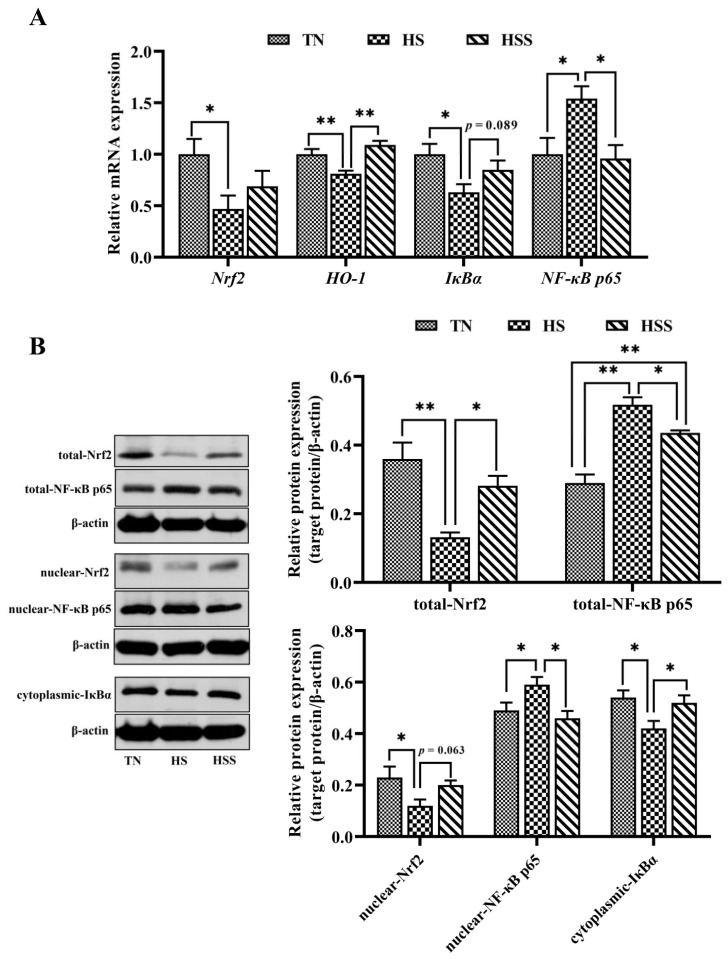
mRNA and protein expression of molecules related to Nrf2 and NF-κB signaling pathways (**A**) after subjecting to heat stress and treatment with dietary seaweed-derived polysaccharides. * *p* < 0.05, ** *p* < 0.01, no superscript marks indicated that *p* > 0.10. The abbreviations for the detected parameters (Nrf2, HO-1, IκBα and NF-κB p65) (**B**) are nuclear factor erythroid 2-related factor 2; heme oxygenase-1; inhibitor kappa B alpha, and nuclear factor-kappa B p65, respectively.

The relative mRNA and protein expression of Nrf2 and NF-κB p65 signaling pathway–related molecules are presented in Figure 7. HS exposure resulted in lower *Nrf2*, *HO-1,* and inhibitor kappa B alpha (*IκBα)* mRNA expression and higher *NF-κB p65* mRNA expression in the spleen (*p* < 0.05). However, dietary SDP improved *HO-1* mRNA expression (*p* < 0.01) but decreased *NF-κB p65* mRNA expression (*p* < 0.05) in the spleen. In addition, the spleens from the HS group exhibited lower total and nuclear Nrf2 and cytoplasmic IκBα protein expression, but higher total and nuclear NF-κB p65 protein expression (*p* < 0.05). In contrast, SDP supplementation improved total Nrf2 protein levels (*p* < 0.05) but reduced total and nuclear NF-κB p65 protein levels (*p* < 0.05) in the spleen from the HS group.

## 3. Discussion

Of late, the average temperature has been gradually rising worldwide. An increase in temperature will adversely affect human health and have a detrimental influence on animals. Thus, high ambient temperature–induced HS has become an environmental hazard and is associated with several negative effects [1,2,3]. Additionally, the warming of the global climate directly affects the marine ecosystem, which has detrimental impacts on seaweeds in terms of their physiology, reproduction, growth, and survival [25]. On the other hand, due to the eutrophication of the oceans caused by human activities, large-scale and frequent outbreaks of green tides occur in the coastal waters of many countries; among them, the survival of EP has not been affected by the increase in ambient temperature and has grown in large quantities since 2008, producing green tides annually in the coastal waters of China [26]. Therefore, EP not only has sufficient sources, the utilization of EP is also urgently needed in order to prevent green tides, such as developing the high-value products from EP for humans and animals. In this context, we evaluated the chemical composition of SDP from EP, and its beneficial function in a chicken HS model. The SDP was obtained through enzymatic extraction and purification. Compared with an earlier study, the content of polysaccharides was higher in our study, which is beneficial in improving the biological activity of SDP [27]. Mw and Mn are commonly used to characterize the molecular weight of polymers. In the current study, the Mw/Mn ratio was 1.805, suggesting that SDP had a uniform molecular weight distribution and a monodisperse polysaccharide, thereby being an ideal polymer [28]. Moreover, the main troughs of wave seen in the FT-IR spectra were in agreement with the typical characteristics of polysaccharides, and the spectral characteristics of SDP were similar to those of EP polysaccharides reported previously [17]. Eleven monosaccharides were identified using HPLC in this study, and the monosaccharides composed of GlcN, Glc, GalA, Man, Xyl, Gal, Ara, GlcA, Fuc, GalN, and Rib in a molar ratio of 5.08:2.77:1.17:0.28:0.26:0.20:0.09:0.08:0.03:0.02:0.02. However, our findings contradicted those previous reports. For instance, Shi et al. [16] have suggested that EP-derived polysaccharides contain 5 monosaccharides, namely, rhamnose (Rha), Glc, Xyl, Man, and Gal, in a molar ratio of 3.2:1:0.97:0.26:0.13. Zhou et al. [20] found that EP polysaccharides are composed of Rha, Glc, and Xyl in a molar ratio of 3.6:1.2:1. Guo et al. [21] indicated that the polysaccharides from EP were composed of 6 monosaccharides, wherein the molar ratio of Rha, GlcA, Ara, Fuc, Xyl, and Glc was 5.12:1.32:3.38:1.62:1:1.03. Differences in the monosaccharide composition of SDP could be due to the geographical area of collection and/or the extraction process of polysaccharides from EP [15]. SDP used in our study was of relatively high purity and rich in monosaccharides, thereby indicating potential scientific significance for further therapeutic studies.

Previous studies have demonstrated that HS exposure could decrease the relative spleen weight [5,8,29], and this impairment could be mitigated by the dietary supplementation of functional polysaccharides (mannan-oligosaccharides) [30]. However, in this study, the relative spleen weight was not significantly impacted by HS or dietary SDP. Similar findings have been reported previously [31], which suggests that the relative weight of the spleen is not affected by HS. Inconsistencies among these studies might be ascribed to the HS duration, growth stages, animal strains, as well as the different types of polysaccharides. Moreover, in this study, we found that HS caused histological damage to the spleen. Consistent with our findings, Hirakawa et al. [5] indicated that 14 days of HS exposure resulted in histopathological changes in the spleen. We also found that apoptosis in the spleen was increased due to HS, which was in accordance with the findings in previous studies [14,32]. Excessive ROS are generated when subjected to HS, leading to unfavorable histological changes and alterations in cellular physiology [33]. This might explain the histological disruption and apoptosis of the spleen caused by HS. Interestingly, we found that dietary SDP supplementation attenuated HS-induced histological damage. Similarly, the existing literature reveals that the use of dietary phytochemicals can improve the morphology of immune organs of animals exposed to HS [10,34]. Previous studies have demonstrated that dietary algae (EP) polysaccharides can alleviate histopathological alterations of immune organs in a stressed animal model [21,24]. The protective effect of SDP on splenic morphology can be likely attributed to the antioxidant and/or anti-inflammatory properties of SDP [15,35]. However, it should be noted that the splenic apoptosis in TN and HS groups is 3.5% and 6.6%, respectively. Additionally, several reports found that different stressors increase the apoptosis of the spleen from approximately 4% (control group) to 6% (stressed group) in chickens [36,37,38]. Zhao et al. [39] showed that the apoptosis of the spleen in the control group was 0.5%, and the stress induced by the toxin only increased the apoptosis of the spleen to 2% in chickens. The present and previous findings suggest that the apoptosis of the spleen is not apparent under normal physiological conditions; even under stress situations, still more than 90% splenocytes were not affected in the chicken model. Furthermore, dietary SDP only slightly reduced the apoptosis of the spleen by 1.3% from the HS group (6.6% in HS vs. 5.3% in HSS, no significant effect). Similar results were obtained in earlier studies on marine polysaccharides attenuating apoptosis of immune organs in chickens [40,41]. Therefore, the development of high-value seaweed products with stronger anti-apoptotic activity in the chicken model requires further research.

Redox balance is critical in maintaining normal cellular physiological status [42]. The redox balance indicates a balance between antioxidant enzymes and free radicals. Nrf2 is a key signaling pathway that activates antioxidant enzymes to promote free radical scavenging [43]. To elucidate the molecular mechanism of the amelioration of HS-induced splenic injury by dietary SDP, the redox balance and Nrf2 signaling pathway were investigated. We found that HS exposure triggered oxidative stress and inhibited the antioxidant enzyme system of the spleen, and that it was related to the suppression of the Nrf2 signaling pathway. The inhibition of Nrf2 and gene expression of antioxidant enzymes have also been demonstrated by previous studies and were in accordance with our findings. These changes probably occurred because chronic HS destroyed the antioxidant system [11,44,45]. Nrf2 is a key cytoprotective transcription factor related to the regulation of oxidative stress through modulating the expression of the genes of the downstream antioxidant enzymes, such as glutathione peroxidase (GSH-Px), HO-1, GST, SOD, and catalase (CAT) [46,47,48]. Numerous studies have attempted to suppress oxidative stress by enhancing Nrf2 expression, and it is interesting to observe that the inclusion of dietary phytochemicals, including polysaccharides, polyphenols, and flavonoids, could improve redox balance status via upregulating the Nrf2 signaling pathway, thereby eliminating ROS and alleviating oxidative stress [11,45,49,50,51]. Furthermore, several studies have demonstrated that multiple naturally occurring polysaccharides improved the antioxidant capacity in animal models [52,53,54,55,56]. In a recent study, Guo et al. [21] used a rodent model and illustrated that the inclusion of dietary SDP could improve the antioxidant enzyme activity. They also confirmed that dietary SDP could prevent oxidative stress and act as a free radical scavenger, which was associated with the activation of the Nrf2 signaling pathway. Additionally, a recent study has reported that dietary SDP promotes antioxidant performance and ameliorates oxidative stress–mediated alteration of the Nrf2 signaling pathway [24]. Similarly, our findings showed that SDP could be used as a modifier of Nrf2 to improve the activity and gene expression of antioxidant enzymes and reduce MDA levels, thus ameliorating HS exposure–induced oxidative stress of the spleen. Noteworthily, there were four antioxidant enzyme-related genes (*SOD2, GPx1, GSTT1,* and *GSTO1*) whose expression was adversely affected by HS exposure, but only two of them (*SOD2* and *GSTO1*) were significantly reverted after feeding SDP. Likewise, other evidence supported that the natural polysaccharides regulate only certain antioxidant genes in chickens [13,57] and rats [58]. Huang et al. [59] demonstrated that a dietary low dose of plant-derived polysaccharides only improved the mRNA expression of jejunal *SOD2* and *GSTP1* and increasing the polysaccharides intake could significantly upregulate the mRNA expression of *SOD1, SOD2, GSTP1,* and *GSTM1* in a mice model of aging. Therefore, it is reasonable to speculate that the impacts of SDP treatment on antioxidant-related genes expression in the spleen may be dose-dependent. However, the specific mechanisms are yet to be elucidated. Regarding the structure-activity relationship between SDP and protection against oxidative stress, on the one hand, the unsaturated bonds in the SDP structure can directly capture and scavenge free radicals [17]; moreover, the functional group of the SDP can competitively bind with regulatory elements such as phosphorylation and ubiquitination molecules, thereby modulating Nrf2 signaling and enhancing antioxidant capacity [15]. In short, the structure-antioxidant activity relationship of polysaccharides is a complex issue, but this study presents the basis for promising future studies.

It has been suggested that the overproduction of ROS under HS conditions also leads to an inflammatory response [48]. NF-κB is involved in the inflammatory response in animals exposed to HS [60,61]. In the physiological resting conditions, most of NF-κB p65 molecules are not in a free state; they are present in combination with IκBα proteins in the cytoplasm [62]. Stress can cause the degradation of IκBα, leading to numerous conversions of NF-κB p65 to its free, activated form. The excessive released NF-κB p65 undergoes nuclear translocation, thus modulating the inflammatory process [60,62,63]. The secretion and expression of cytokines are related to T-helper (Th) cells. IL-2 and IFN-γ are mainly secreted by Th1 cells; and IL-1β, IL-4, IL-6, and IL-10 are mainly secreted by Th2 cells [64]. Furthermore, the Th1/Th2 balance plays an important role in immune response. Emerging evidence suggests that extreme environmental factors can cause inflammation of immune organs by disturbing the Th1/Th2 balance and activating the NF-κB p65 signaling pathway [65,66,67]. We found that HS elevated TNF-α, IL-4 and IL-10 levels (including the corresponding genes expression), reduced INF-γ and IL-2 levels (*INF-γ* expression) and promoted cytoplasmic IκBα degradation and nuclear expression of NF-κB p65 in the spleen. Similarly, He et al. [10] reported that HS triggered a Th1/Th2 imbalance and activated the NF-κB signaling pathway in the spleen; their findings were based on the mRNA expression of cytokines and IκBα/NF-κB p65. Keeping in mind the anti-inflammatory activity of natural polysaccharides, we sought to further clarify how the administration of dietary SDP could alleviate the HS-induced inflammatory response. It was found that dietary SDP attenuated the inflammatory response in the spleen by inhibiting the activation of NF-κB p65 when exposed to HS. Similar to the findings in our study, it has been suggested that SDP from EP could improve the immune response in fish [20] and mice [18] via the regulation of the NF-κB signaling pathway. Liu et al. [68] demonstrated that dietary *Astragalus* polysaccharides (APS) improve the Th1/Th2 balance by suppressing *NF-κB p65* expression in the jejunal mucosa of animals challenged with lipopolysaccharide. Hu et al. [52] observed that the polysaccharides from *Agaricus blazei* mitigated hepatic inflammation by downregulating *IL-1β, TNF-α,* and *IL-6* expression in animals exposed to cadmium. Dong et al. [69] found that the dietary inclusion of APS ameliorated the inflammatory response by upregulating IκBα degradation and downregulating NF-κB p65 activation in a mouse model of stress. Farag et al. [70] reported that APS could alleviate the tilmicosin-induced inflammatory response in mice by inhibiting the NF-κB signaling pathway. Although this evidence showed that the seaweed- and plant-derived polysaccharides could protect immune organs against inflammation and it was related to the suppression of NF-κB signaling, it has also been demonstrated that NF-κB is not only involved in the regulation of inflammation. The NF-κB p65 can form hetero- and homodimers of different composition and bind to a variety of DNA sequences called κB sites, thus effectively regulating diverse target genes [71]. Indeed, it has been proven that the activation of NF-κB is an evolutionarily conserved and efficient mechanism for maintaining physiological homeostasis and immunity in the host [72]. Under normal status, the majority of NF-κB p65-activated genes regulate biological processes associated with cell growth, protection, and repair, etc. [73]. However, excessive NF-κB activation in pathophysiological conditions leads to detrimental consequences such as chronic inflammation [62,74]. Thus, in our study, the relatively prominent Western blotting (WB) bands of nuclear NF-κB p65 appeared in the TN group (the relative expression values are between 0.4–0.5), possibly due to the fact that chickens are farm animals with exuberant metabolism and growth rates; the nuclear translocated NF-κB p65 mainly plays a role in regulating biological processes such as cell growth and differentiation in avian species under physiological conditions [75]. Likewise, Cheng et al. [76] and Tong et al. [77] demonstrated that the hepatic nuclear NF-κB p65 shows a strong expression signal in the control chicken group, which has a similar relative expression level with the current study. On the other hand, our study also observed significant WB bands of cytoplasmic IκBα in the TN group; this is similar to the results of earlier reports [10,77]. It appears to contradict the findings of nuclear translocation of p65 in the TN group. This is due to the fact that IκBα/NF-κB p65 signaling is mainly associated with the feedback regulation of immune responses [74]. The nuclear translocation of NF-κB p65 is also modulated by other means than the degradation of IκBα, such as the cleavage of the inhibitory ankyrin repeat domains of NF-κB subunits (p100 and p105), as a result of responsible for the biological systems (e.g., cell growth, enzyme synthesis) [71,75]. This may well explain the phenomenon that we observed the relationship between nuclear NF-κB p65 and cytoplasmic IκBα expression in chickens under normal conditions. Furthermore, another interesting observation that needs attention is that the effects of SDP treatment on the protein and mRNA expression of inflammatory cytokines are inconsistent. Dietary SDP significantly affected the TNF-α, IL-2, IL-4, and IL-10 contents, but only reduced the mRNA expression of *IL-4* in the spleen when exposed to HS. It is well known that there is a gap between mRNA and protein expression, previous studies also found that the natural polysaccharides show differential regulatory effects in the mRNA and protein expression of cytokines in mice and chickens [69,78]. The mRNA expression is a part of the indicators in our study, and the protein expression proved that SDP treatment has significant impacts on inflammatory cytokines of the spleen after being subjected to HS. Therefore, with an integrated analysis of the results of inflammatory response, we can conclude that dietary SDP attenuates HS-induced inflammatory response through downregulating the degradation of IκBα and suppressing the excessive nuclear translocation of NF-κB p65. However, in view of the complexity of NF-κB regulation, in-depth molecular studies are essential to elucidate the mechanism of action of dietary SDP with respect to the NF-κB signaling pathway.

## 4. Materials and Methods

### 4.1. Preparation, Chemical Composition, and Structure Analysis of SDP

#### 4.1.1. Preparation of SDP

The SDP used in this study was derived from natural green seaweed (EP, collected offshore Qingdao, China, Haida Biotechnology Co., Ltd., Qingdao, China), which is rich in water-soluble sulfated polysaccharides. Enzymatic hydrolysis, purification, concentration, and spray drying were used for the preparation of SDP. Briefly, the collected seaweed was pulverized using ultrasonication and the powdered seaweed was soaked in hot water for extraction. Subsequently, the aqueous extracts of the seaweed were subjected to stepwise enzymatic degradation using pectinase, cellulase, etc. SDP was obtained after the steps of enzyme inactivation, centrifugal concentration, column separation using gel chromatography, fractional precipitation using ethanol, and spray drying.

#### 4.1.2. Assay to Determine the Chemical Composition of SDP

The phenol sulfuric acid assay was used to determine the total polysaccharide content using Glc as a standard. Briefly, 0.2, 0.4, 0.6, 0.8, 1.0, and 1.2 mL of Glc standard solution were taken and 2 mL of distilled water was added. Next, 5 mL of sulfuric acid and 2.0 mL of phenol solution (5%) were added, and the reaction mixture was incubated in a water bath at 100 oboC for 15 min. The absorbance was detected at 490 nm (SP-752 UV-VIS spectrophotometer, Spectrum Instrument Co., Ltd., Shanghai, China) and a Glc standard curve was prepared accordingly. The absorbance of SDP samples was measured at a wavelength of 490 nm. Lastly, the total polysaccharide concentration (in terms of Glc) was determined using the standard curve. Bradford’s method was used to measure the protein content of SDP using BSA as a standard. Sulfate content was detected using barium sulfate-gelatin turbidimetry with potassium sulfate as a standard. m-Hydroxybiphenyl colorimetry was used to determine the uronic acid content and d-GlcA was used as a standard. Assay procedures and details on determining the protein, sulfate, and uronic acid contents were based on a previous study [27]. The moisture content was determined using the drying method and the assay procedures were carried out according to AOAC (Association of Official Analytical Chemists, 2012) [79].

#### 4.1.3. Determination of the Molecular Weight of SDP

Gel-permeation chromatography (GPC) was used to analyze the molecular weight of SDP. GPC was conducted in conjunction with an HPLC system (Agilent 1200, Palo Alto, CA, USA). An Ultrahydrogel^TM^ Linear (300 mm × 7.8 mm. Waters., Palo Alto, CA, USA) gel-filtration chromatographic column was used for GPC. The molecular weight of SDP was estimated using a standard curve for dextran. Data of the molecular weight, including Mn, Mw, Mp, and Mz, were collected and analyzed using Astra software (v7.0, Wyatt Technologies, Inc., Santa Barbara, CA, USA). Former reports were referred to for the detailed GPC procedures and the definitions of Mn, Mw, Mp, and Mz [17,28].

#### 4.1.4. Assay to Determine the Monosaccharide Composition of SDP

The monosaccharide composition of SDP was measured using HPLC. An Agilent 1200 HPLC system (Palo Alto, CA, USA) coupled with a SHISEIDO C18 column (250 mm × 4.6 mm, Palo Alto, CA, USA) was used. Monosaccharide standards, including those for Man, Rib, Rha, Fuc, GlcN, Xyl, Ara, Gal, GalN, Glc, GalA, and GlcA, were obtained commercially (Sigma-Aldrich chemical Co. Milwaukee, WI, USA). Analytical procedures were based on previous study [78].

#### 4.1.5. Fourier-Transform Infrared (FT-IR) Spectroscopy

The preliminary structure of SDP was determined using FT-IR. The SDP sample was dried prior to tableting with KBr powder and subsequently analyzed in the wavenumber range of 4000–500 cm^−1^ using a Nicolet^TM^ 6700 FT-IR spectrometer (Thermo Fisher Scientific Co., Ltd., Waltham, MA, USA) and a standard procedure to prepare a KBr pellet [80].

### 4.2. Animals, Diet, and Experimental Design

Eight-week-old indigenous male broilers (Huaixiang chickens; Guangdong local hatchery, Maoming, China) were used for the animal study. The choice of animals was based on a former study [81]. In total, 144 chickens were randomly assigned to one of three treatment groups: TN group (raised under 23.6 ± 1.8 °C as thermoneutral); HS group (raised under 33.2 ± 1.5 °C, 10 h/day, 8:00 am to 6:00 pm); and HSS group (HS group supplemented 1000 mg/kg SDP). Each treatment group was subjected to six replicates (eight broilers/replicate). Relative humidity in all groups was maintained in the range of 55–75%. The animal experiment was conducted for four weeks. We referred to our earlier study for monitoring the environmental conditions and determining the diet composition [2]. The form of the diet was mash, and the diet formulation was based on the feeding standard [82]. All animals were provided access to clean water and food ad libitum.

### 4.3. Sampling

After completion of the intervention, one chicken from each replicate cage was randomly selected for slaughter by neck bloodletting and sampling (n = 6/treatment). Before slaughter, the selected chickens were individually weighed. After slaughter, the spleens were quickly separated, rinsed with saline, and weighed. Next, 10% neutral-buffered formalin was used to process spleen samples for histological studies and to determine cell apoptosis. Unused spleen tissues were stored at −80 °C until further biochemical and molecular analyses.

### 4.4. Histology, Apoptosis and Relative Weight of Spleen

For histological analysis, spleen samples were fixed in 10% neutral-buffered formalin for 48 h. Next, the samples were embedded in paraffin, cut into 5-μm-thick sections, and stained using hematoxylin and eosin [83]. An SDPTOP (GD-30RFL) optical microscope (Guangzhou, China) was used to observe slides under 400× magnification. TCapture Imaging Application 4.3 software (Guangzhou, China) was used to obtain images and analyze the histopathological changes. The observation and analysis of histopathological changes of the spleen were based on the previous studies [38,39].

The TdT-mediated dUTP Nick-End Labeling (TUNEL) method was used to determine cell apoptosis using the paraffinized spleen sections. Primary reagents include the TUNEL assay kit (Roche, 11684817910, Basel, Switzerland), Antifade Mounting Medium (Servicebio, G1401, Wuhan, China) and drip-4′,6-diamidino-2-phenylindole (DAPI) (Servicebio, G1012, Wuhan, China). Samples were analyzed following the manufacturer’s instructions and based on a former study [84]. Fluorescence microscopy (Nikon Eclipse, C1, Tokyo, Japan) was used to observe the spleen sections, and Image-Pro Plus 6.0 (Rockville, MD, USA) was used to acquire and analyze images (200× magnification). Apoptosis-positive cells appear green in the figures. The apoptosis rate (%) was calculated as follows: (positive apoptotic cells/total cells) × 100%.

The relative weights of spleens were calculated as follows: (spleen weight [g]/body weight [g]) × 100%.

### 4.5. Determination of Antioxidant Parameters and Cytokine Concentrations

The antioxidant system–associated parameters of the spleen, including T-AOC; GSH-Px, T-SOD, GST, and CAT activities; and MDA and ROS levels, were determined using the respective commercial kits from Jiancheng Institute (Nanjing, China) following the manufacturers’ instructions, and the catalog number of each kit as follows: T-AOC (A015-2-1), GSH-Px (A005-1-2), T-SOD (A001-3-2), GST (A004-1-1), CAT (A007-1-1), MDA (A003-1-2), and ROS (E004-1-1). The cytokine concentrations of splenic samples, including TNF-α, IL-1β, IL-2, IL-4, IL-6, IL-10, and IFN-γ were determined using the corresponding ELISA kits from Jiangsu Enzyme Immunology Co., Ltd. (Suzhou, China) following the manufacturers’ instructions (Catalog number: TNF-α, MM-0938O2; IL-1β, MM-36910O2; IL-2, MM-0528O2; IL-4, MM-0527O2; IL-6, MM-0521O2; IL-10 MM-1145O2; IFN-γ, MM-0520O1).

### 4.6. Quantitative Real-Time Polymerase Chain Reaction (qPCR) to Analyze Gene Expression

Total RNA extraction and cDNA transcription protocols used to process the spleen samples are described in our earlier report [2]. The main reagents included RNA extraction kits from Nanjing Jiancheng Institute (Nanjing, China) and an RT reagent kit (TaKaRa Biotechnology Co., Ltd., Beijing, China). After the preparation of cDNA, qPCR was used to determine mRNA expression level. The *β-actin* was used as an internal control gene in the spleen of chickens according to the earlier study [79], and we also found that the expression of *β-actin* gene in the chicken spleen was stable through pre-experimental evaluation. We referred to our previous study for primer information and detailed qPCR reaction conditions [2]. Each splenic sample was tested in triplicate. The 2^−ΔΔCt^ method was used for data processing and the results are presented as relative mRNA expression compared with the TN group [85].

### 4.7. Western Blotting

Western blotting was used to determine protein expression levels in spleen samples. Detailed methods of analysis and information on Western blotting have been described previously [2,24]. Details of the primary kits and antibodies are as follows: (1) total, nuclear and cytoplasmic protein extraction kits were obtained from Servicebio Technology Co., Ltd. (Wuhan, China); (2) primary antibody against Nrf2 (rabbit polyclonal antibody, 16396-1-ap, 68 kDa, dilution 1:1000) from Servicebio; (3) primary antibody against NF-κB p65 (rabbit polyclonal antibody, GB11997, 65 kDa, dilution 1:1000) from Servicebio; (4) primary antibody against IκBα (rabbit polyclonal antibody, A1096, 36 kDa, dilution 1:1000) from Beyotime Biotechnology Co., Ltd. (Shanghai, China); (5) goat anti-rabbit IgG and with HRP-labeled secondary antibodies at a dilution of 1:3000 from Servicebio; and (6) β-actin was used as the internal control and with rabbit IgG polyclonal antibody (dilution 1:3000). Alpha Imager from Alpha Innotech Co., Ltd. (California, CA, USA) was used to analyze the protein bands. Results are expressed as relative protein expression: the ratio of the target protein to β-actin.

### 4.8. Statistical Analysis

Data were analyzed using the general linear model procedure in SAS 9.4 (SAS, 2013. SAS Institute Inc., Cary, NC, USA). Tukey’s test was used for comparing differences among groups (TN vs. HS, HS vs. HSS, and TN vs. HSS). A *P*-value between 0.05 and 0.10 was considered to be a trend of significance. *p* < 0.05 was considered statistically significant.

## 5. Conclusions

**Figure 8:**
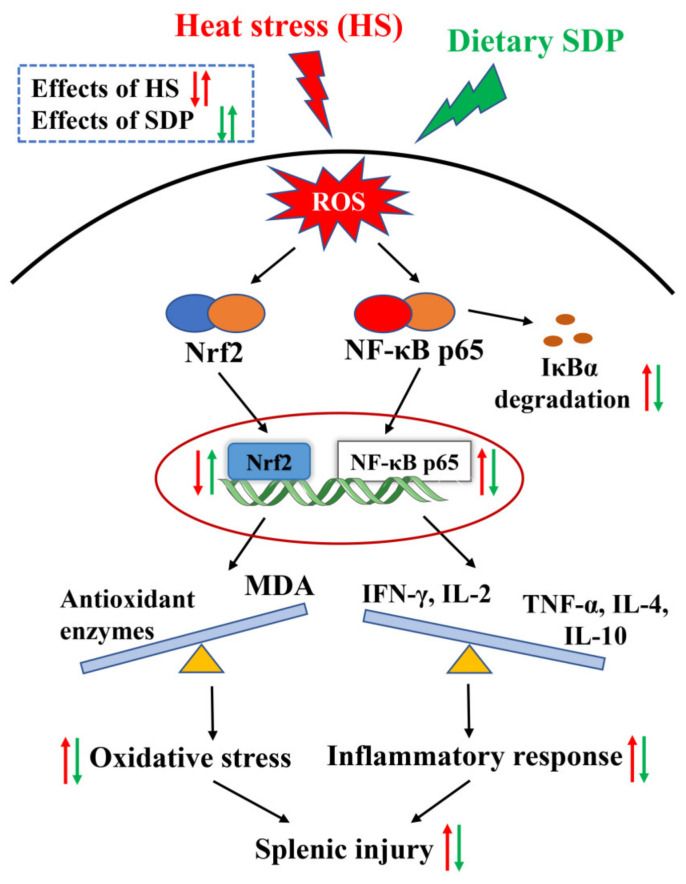
Proposed model of dietary inclusion of seaweed-derived polysaccharides attenuating heat stress–induced splenic oxidative stress and inflammatory response. SDP, seaweed-derived polysaccharides; ROS, reactive oxygen species; IκBα, inhibitor kappa B alpha; Nrf2, nuclear factor erythroid 2-related factor 2; NF-κB p65, nuclear factor-kappa B p65; MDA, malondialdehyde; TNF-α, tumor necrosis factor-α; IFN-γ, interferon-γ; IL interleukin.

The innovative findings of this study reveal that dietary SDP from EP ameliorates HS-induced oxidative stress and inflammatory responses in the spleen. The protective effect of SDP is likely associated with the activation of the Nrf2 signaling pathway and the suppression of the NF-κB signaling pathway (the proposed model of SDP was summarized in Figure 8). The results provide the mechanistic basis for the use of SDP in alleviating HS-induced splenic damage. Moreover, it serves as a reference for the use of SDP as an antioxidant and immune enhancer, indicating its potential to be further developed as a marine drug.

## Figures and Tables

**Table 1:** Chemical composition of seaweed-derived polysaccharides (SDP) from *Enteromorpha prolifera*.

Items	Contents, %
Total polysaccharides	53.32
Sulfate	19.87
Uronic acid	12.66
Moisture	3.58
Protein	1.65
Unspecified compounds (lipid, salt, etc.)	8.92

**Table 2:** Monosaccharide composition of seaweed-derived polysaccharides (SDP) from *Enteromorpha prolifera*.

Items	Contents, %
Glucosamine (GlcN)	50.81
Glucose (Glc)	27.70
Galacturonic acid (GalA)	11.75
Mannose (Man)	2.78
Xylose (Xyl)	2.63
Galactose (Gal)	1.95
Arabinose (Ara)	0.93
Glucuronic acid (GlcA)	0.76
Fucose (Fuc)	0.27
Galactosamine (GalN)	0.25
Ribose (Rib)	0.17

**Table 3:** Antioxidant-related parameters of the spleen after subjecting to heat stress and treatment with dietary seaweed-derived polysaccharides.

Items	Units	TN	HS	HSS	*p*-Value		
					TN vs. HS	HS vs. HSS	TN vs. HSS
T-AOC	mmol/mg protein	36.36 ± 1.39	23.91 ± 2.37	32.10 ± 1.70	<0.001	0.010	0.132
T-SOD	U/mg protein	63.12 ± 2.79	46.21 ± 4.14	54.90 ± 4.28	0.029	0.218	0.242
GSH-Px	U/mg protein	126.39 ± 8.02	130.06 ± 7.86	125.28 ± 4.02	0.709	0.627	0.908
CAT	U/mg protein	3.62 ± 0.46	3.38 ± 0.39	3.15 ± 0.37	0.698	0.746	0.481
GST	U/mg protein	42.00 ± 1.96	26.42 ± 1.41	38.39 ± 2.00	<0.001	0.002	0.223
MDA	nmol/mg protein	2.28 ± 0.15	4.12 ± 0.30	2.95 ± 0.43	0.019	0.045	0.211
ROS	fluorescence/mg protein	92.98 ± 3.04	131.75 ± 10.91	114.50 ± 6.18	0.016	0.225	0.138

The data are presented as mean ± standard error. TN, thermoneutral zone; HS, heat stress; HSS, HS group fed a diet containing 1000 mg/kg seaweed-derived polysaccharides. The abbreviations for T-AOC, T-SOD, GSH-Px, CAT, GST, MDA, and ROS are total antioxidant capacity; total superoxide dismutase; glutathione peroxidase; catalase; glutathione-S transferase; malondialdehyde and reactive oxygen species, respectively.

**Table 4:** Cytokine concentrations of the spleen after subjecting to heat stress and treatment with dietary seaweed-derived polysaccharides.

Items	Units	TN	HS	HSS	*p*-Value		
					TN vs. HS	HS vs. HSS	TN vs. HSS
TNF-α	pg/mg protein	203.68 ± 5.91	252.65 ± 10.38	218.10 ± 5.10	0.001	0.011	0.224
IFN-γ	pg/mg protein	421.19 ± 9.87	377.94 ± 7.88	407.04 ± 10.96	0.024	0.103	0.404
IL-1β	pg/mg protein	310.97 ± 9.60	338.40 ± 9.48	320.76 ± 10.08	0.141	0.329	0.581
IL-2	ng/mg protein	10.38 ± 0.41	8.25 ± 0.27	10.16 ± 0.33	<0.001	0.001	0.648
IL-4	ng/mg protein	72.69 ± 6.09	113.72 ± 7.45	81.12 ± 1.76	<0.001	<0.001	0.853
IL-6	pg/mg protein	186.48 ± 6.81	207.26 ± 9.46	198.05 ± 2.80	0.126	0.475	0.376
IL-10	ng/mg protein	181.22 ± 7.83	224.09 ± 12.62	190.46 ± 6.71	0.008	0.026	0.489

The data are presented as mean ± standard error. TN, thermoneutral zone; HS, heat stress; HSS, HS group fed diet contained 1000 mg/kg seaweed-derived polysaccharides; SEM, standard error of the means. The abbreviations for the detected parameters (TNF-α, IFN-γ and IL) are tumor necrosis factor-α, interferon-γ and interleukin, respectively.

## Data Availability

The data supporting the conclusions of this article will be made available by the authors, without undue reservation.

## References

[1] Chauhan S.S., Rashamol V., Bagath M., Sejian V., Dunshea F.R. (2021). Impacts of heat stress on immune responses and oxidative stress in farm animals and nutritional strategies for amelioration. Int. J. Biometeorol..

[2] Liu W.C., Ou B.H., Liang Z.L., Zhang R., Zhao Z.H. (2021). Algae-derived polysaccharides supplementation ameliorates heat stress-induced impairment of bursa of Fabricius via modulating NF-κB signaling pathway in broilers. Poult. Sci..

[3] Rhoads R.P., Baumgard L.H., Suagee J.K., Sanders S.R. (2013). Nutritional interventions to alleviate the negative consequences of heat stress. Adv. Nutr..

[4] Emami N.K., Jung U., Voy B., Dridi S. (2021). Radical Response: Effects of heat stress-induced oxidative stress on lipid metabolism in the avian liver. Antioxidants.

[5] Hirakawa R., Nurjanah S., Furukawa K., Murai A., Kikusato M., Nochi T., Toyomizu M. (2020). Heat stress causes immune abnormalities via massive damage to effect proliferation and differentiation of lymphocytes in broiler chickens. Front. Vet. Sci..

[6] Guo Y., Balasubramanian B., Zhao Z.H., Liu W.C. (2021). Heat stress alters serum lipid metabolism of Chinese indigenous broiler chickens-a lipidomics study. Environ. Sci. Pollut. Res..

[7] Quinteiro-Filho W.M., Ribeiro A., Ferraz-de-Paula V., Pinheiro M., Sakai M., Sá L., Ferreira A., Palermo-Neto J. (2010). Heat stress impairs performance parameters, induces intestinal injury, and decreases macrophage activity in broiler chickens. Poult. Sci..

[8] Chegini S., Kiani A., Rokni H. (2018). Alleviation of thermal and overcrowding stress in finishing broilers by dietary propolis supplementation. Ital. J. Anim. Sci..

[9] Ohtsu H., Yamazaki M., Abe H., Murakam H., Toyomizu M. (2015). Heat stress modulates cytokine gene expression in the spleen of broiler chickens. J. Poult. Sci..

[10] He S., Yu Q., He Y., Hu R., Xia S., He J. (2019). Dietary resveratrol supplementation inhibits heat stress-induced high-activated innate immunity and inflammatory response in spleen of yellow-feather broilers. Poult. Sci..

[11] Sahin K., Orhan C., Tuzcu M., Sahin N., Hayirli A., Bilgili S., Kucuk O. (2016). Lycopene activates antioxidant enzymes and nuclear transcription factor systems in heat-stressed broilers. Poult. Sci..

[12] Awais M.M., Akhtar M., Anwar M.I., Khaliq K. (2018). Evaluation of Saccharum officinarum L. bagasse-derived polysaccharides as native immunomodulatory and anticoccidial agents in broilers. Vet. Parasitol..

[13] Wassie T., Duan X., Xie C., Wang R., Wu X. (2022). Dietary Enteromorpha polysaccharide-Zn supplementation regulates amino acid and fatty acid metabolism by improving the antioxidant activity in chicken. J. Anim. Sci. Biotechnol..

[14] Wassie T., Lu Z., Duan X., Xie C., Gebeyew K., Yumei Z., Wu X. (2021). Dietary Enteromorpha polysaccharide enhances intestinal immune response, integrity, and caecal microbial activity of broiler chickens. Front. Nutr..

[15] Zhong R., Wan X., Wang D., Zhao C., Liu D., Gao L., Wang M., Wu C., Nabavid S.M., Daglia M. (2020). Polysaccharides from marine Enteromorpha: Structure and function. Trends Food Sci. Technol..

[16] Shi M.J., Wang F., Jiang H., Qian W.W., Xie Y.Y., Wei X.Y., Zhou T. (2020). Effect of enzymatic degraded polysaccharides from Enteromorpha prolifera on the physical and oxidative stability of fish oil-in-water emulsions. Food Chem..

[17] Shi M.J., Wei X., Xu J., Chen B.J., Zhao D.Y., Cui S., Zhou T. (2017). Carboxymethylated degraded polysaccharides from Enteromorpha prolifera: Preparation and in vitro antioxidant activity. Food Chem..

[18] Wei J., Wang S., Liu G., Pei D., Liu Y., Liu Y., Di D. (2014). Polysaccharides from Enteromorpha prolifera enhance the immunity of normal mice. Int. J. Biol. Macromol..

[19] Zhao Y., Balasubramanian B., Guo Y., Qiu S.J., Jha R., Liu W.C. (2021). Dietary Enteromorpha polysaccharides supplementation improves breast muscle yield and is associated with modification of mRNA transcriptome in broiler chickens. Front. Vet Sci..

[20] Zhou Z., Pan S., Wu S. (2020). Modulation of the growth performance, body composition and nonspecific immunity of crucian carp Carassius auratus upon Enteromorpha prolifera polysaccharide. Int. J. Biol. Macromol..

[21] Guo F., Zhuang X., Han M., Lin W. (2020). Polysaccharides from Enteromorpha prolifera protect against carbon tetrachloride-induced acute liver injury in mice via activation of Nrf2/HO-1 signaling, and suppression of oxidative stress, inflammation and apoptosis. Food Funct..

[22] Guo Y., Zhao Z.H., Pan Z.Y., An L.L., Balasubramanian B., Liu W.C. (2020). New insights into the role of dietary marine-derived polysaccharides on productive performance, egg quality, antioxidant capacity, and jejunal morphology in late-phase laying hens. Poult. Sci..

[23] Liu W.C., Guo Y., Zhao Z.H., Jha R., Balasubramanian B. (2020). Algae-derived polysaccharides promote growth performance by improving antioxidant capacity and intestinal barrier function in broiler chickens. Front. Vet. Sci..

[24] Guo Y., Balasubramanian B., Zhao Z.H., Liu W.C. (2021). Marine algal polysaccharides alleviate aflatoxin B1-induced bursa of Fabricius injury by regulating redox and apoptotic signaling pathway in broilers. Poult. Sci..

[25] Harley C.D., Anderson K.M., Demes K.W., Jorve J.P., Kordas R.L., Coyle T.A., Graham M.H. (2012). Effects of climate change on global seaweed communities. J. Phycol..

[26] Wang Z., Fu M., Xiao J., Zhang X., Song W. (2018). Progress on the study of the Yellow Sea green tides caused by Enteromorpha prolifera. Acta Oceanol. Sin..

[27] Lin Y. (2009). Studies on Extraction, Purification and Bioactivities of Polysaccharides from Enteromorpha Prolifera. Master’s Thesis.

[28] Hu W. (2012). Polymer Physics: A molecular Approach.

[29] Liu L.L., He J.H., Xie H.B., Yang Y.S., Li J.C., Zou Y. (2014). Resveratrol induces antioxidant and heat shock protein mRNA expression in response to heat stress in black-boned chickens. Poult. Sci..

[30] Sohail M., Ijaz A., Younus M., Shabbir M., Kamran Z., Ahmad S., Anwar H., Yousaf M., Ashraf K., Shahzad A. (2013). Effect of supplementation of mannan oligosaccharide and probiotic on growth performance, relative weights of viscera, and population of selected intestinal bacteria in cyclic heat-stressed broilers. J. Appl. Poult. Res..

[31] Hosseini-Vashan S.J., Raei-Moghadam M.S. (2019). Antioxidant and immune system status, plasma lipid, abdominal fat, and growth performance of broilers exposed to heat stress and fed diets supplemented with pomegranate pulp (*Punica granatum* L.). J. Appl. Anim. Res..

[32] Xu D.N., Li W.Y., Li B.X., Tian Y.B., Huang Y. (2017). The effect of selenium and polysaccharide of Atractylodes macrocephala Koidz. (PAMK) on endoplasmic reticulum stress and apoptosis in chicken spleen induced by heat stress. RSC Adv..

[33] Vandana G.D., Sejian V., Lees A.M., Pragna P., Silpa M.V., Maloney S.K. (2021). Heat stress and poultry production: Impact and amelioration. Int. J. Biometeorol..

[34] Awad E., Zulkifli I., Ramiah S., Khalil E., Abdallh M. (2020). Prebiotics supplementation: An effective approach to mitigate the detrimental effects of heat stress in broiler chickens. World’s Poult. Sci. J..

[35] Liu W.C., Zhou S.H., Balasubramanian B., Zeng F.Y., Sun C.B., Pang H.Y. (2020). Dietary seaweed (Enteromorpha) polysaccharides improves growth performance involved in regulation of immune responses, intestinal morphology and microbial community in banana shrimp Fenneropenaeus merguiensis. Fish Shellfish Immun..

[36] Peng X., Cui H., He Y., Cui W., Fang J., Zuo Z., Lai W. (2012). Excess dietary sodium selenite alters apoptotic population and oxidative stress markers of spleens in broilers. Biol. Trace Elem. Res..

[37] Chen S.S., Li Y.H., Lin M.F. (2017). Chronic exposure to the Fusarium mycotoxin deoxynivalenol: Impact on performance, immune organ, and intestinal integrity of slow-growing chickens. Toxins.

[38] Chen Y., Han S., Wang Y., Li D., Zhao X., Zhu Q., Yin H. (2019). Oxidative stress and apoptotic changes in broiler chicken splenocytes exposed to T-2 toxin. BioMed Res. Inter..

[39] Zhao H., He Y., Li S., Sun X., Wang Y., Shao Y., Hou Z., Xing M. (2017). Subchronic arsenism-induced oxidative stress and inflammation contribute to apoptosis through mitochondrial and death receptor dependent pathways in chicken immune organs. Oncotarget.

[40] Liu W.C., Zhu Y.R., Zhao Z.H., Jiang P., Yin F.Q. (2021). Effects of dietary supplementation of algae-derived polysaccharides on morphology, tight junctions, antioxidant capacity and immune response of duodenum in broilers under heat stress. Animals.

[41] Li X., Ding X., Peng X., Chi X., Cui H., Zuo Z., Fang J. (2017). Effect of chitosan oligosaccharides on antioxidant function, lymphocyte cycle and apoptosis in ileum mucosa of broiler. Kafkas Univ. Vet. Fak. Derg..

[42] Banerjee B.D., Seth V., Bhattacharya A., Pasha S.T., Chakraborty A.K. (1999). Biochemical effects of some pesticides on lipid peroxidation and free-radical scavengers. Toxicol. Lett..

[43] Itoh K., Chiba T., Takahashi S., Ishii T., Igarashi K., Katoh Y., Oyake T., Hayashi N., Satoh K., Hatayama I. (1997). An Nrf2/small Maf heterodimer mediates the induction of phase II detoxifying enzyme genes through antioxidant response elements. Biochem. Biophys. Res. Commun..

[44] Arain M.A., Mei Z., Hassan F., Saeed M., Alagawany M., Shar A., Rajput I. (2018). Lycopene: A natural antioxidant for prevention of heat-induced oxidative stress in poultry. World’s Poult. Sci. J..

[45] Sahin K., Orhan C., Tuzcu M., Borawska M.H., Jabłonski J., Guler O., Sahin N., Hayirli A. (2013). Berberis vulgaris root extract alleviates the adverse effects of heat stress via modulating hepatic nuclear transcription factors in quails. Brit. J. Nutr..

[46] Yamamoto T., Suzuki T., Kobayashi A., Wakabayashi J., Maher J., Motohashi H., Yamamoto M. (2008). Physiological significance of reactive cysteine residues of Keap1 in determining Nrf2 activity. Mol. Cell. Biol..

[47] Itoh K., Wakabayashi N., Katoh Y., Ishii T., Igarashi K., Engel J.D., Yamamoto M. (1999). Keap1 represses nuclear activation of antioxidant responsive elements by Nrf2 through binding to the amino-terminal Neh2 domain. Gene. Dev..

[48] Sahin K. (2015). Modulation of NF-κB and Nrf2 pathways by lycopene supplementation in heat-stressed poultry. World’s Poult. Sci. J..

[49] Cheng Y., Chen Y., Chen R., Su Y., Zhang R., He Q., Wang K., Wen C., Zhou Y. (2019). Dietary mannan oligosaccharide ameliorates cyclic heat stress-induced damages on intestinal oxidative status and barrier integrity of broilers. Poult. Sci..

[50] Sandner G., Mueller A.S., Zhou X., Stadlbauer V., Schwarzinger B., Schwarzinger C., Wenzel U., Maenner K., van der Klis J.D., Hirtenlehner S. (2020). Ginseng extract ameliorates the negative physiological effects of heat stress by supporting heat shock response and improving intestinal barrier integrity: Evidence from studies with heat-stressed Caco-2 cells, *C. elegans* and growing broilers. Molecules.

[51] Zhang J., Bai K., Su W., Wang A., Zhang L., Huang K., Wang T. (2018). Curcumin attenuates heat-stress-induced oxidant damage by simultaneous activation of GSH-related antioxidant enzymes and Nrf2-mediated phase II detoxifying enzyme systems in broiler chickens. Poult. Sci..

[52] Hu X., Zhang R., Xie Y., Wang H., Ge M. (2017). The protective effects of polysaccharides from Agaricus blazei Murill against cadmium-induced oxidant stress and inflammatory damage in chicken livers. Biol. Trace Elem. Res..

[53] Long L., Kang B., Jiang Q., Chen J. (2020). Effects of dietary Lycium barbarum polysaccharides on growth performance, digestive enzyme activities, antioxidant status, and immunity of broiler chickens. Poult. Sci..

[54] Zhang C., Li C., Shao Q., Chen W., Ma L., Xu W., Li Y., Huang S., Ma Y. (2021). Effects of Glycyrrhiza polysaccharide in diet on growth performance, serum antioxidant capacity, and biochemistry of broilers. Poult. Sci..

[55] Zhang S., Wang C., Sun Y., Wang G., Chen H., Li D., Yu X., Chen G. (2018). Xylanase and fermented Polysaccharide of Hericium caputmedusae reduce pathogenic infection of broilers by improving antioxidant and anti-inflammatory properties. Oxid. Med. Cell. Longev..

[56] Zhou M., Tao Y., Lai C., Huang C., Zhou Y., Yong Q. (2019). Effects of mannanoligosaccharide supplementation on the growth performance, immunity, and oxidative status of partridge shank chickens. Animals.

[57] Xing Y.Y., Zheng Y.K., Yang S., Zhang L.H., Guo S.W., Shi L.L., Xu Y.Q., Jin X., Yan S.M., Shi B.L. (2021). Artemisia ordosica polysaccharide alleviated lipopolysaccharide-induced oxidative stress of broilers via Nrf2/Keap1 and TLR4/NF-κB pathway. Ecotox. Environ. Saf..

[58] Xing Y.Y., Xu Y.Q., Jin X., Shi L.L., Guo S.W., Yan S.M., Shi B.L. (2020). Optimization extraction and characterization of Artemisia ordosica polysaccharide and its beneficial effects on antioxidant function and gut microbiota in rats. RSC Adv..

[59] Huang C., Cao X., Chen X., Fu Y., Zhu Y., Chen Z., Luo Q., Li L., Song X., Jia R. (2017). A pectic polysaccharide from Ligusticum chuanxiong promotes intestine antioxidant defense in aged mice. Carbohyd. Polym..

[60] Goel A., Ncho C.M., Choi Y.H. (2021). Regulation of gene expression in chickens by heat stress. J. Anim. Sci. Biotechnol..

[61] Lan X., Hsieh J.C., Schmidt C.J., Zhu Q., Lamont S.J. (2017). Heat stress alters immune pathways in liver of divergent chicken lines. Anim. Indust. Rep..

[62] Baldwin A.S. (1996). The NF-κB and IκB proteins: New discoveries and insights. Annu. Rev. Immunol..

[63] Chi Q., Wang D., Hu X., Li S., Li S. (2019). Hydrogen sulfide gas exposure induces necroptosis and promotes inflammation through the MAPK/NF-κB pathway in broiler spleen. Oxid. Med. Cell. Longev..

[64] Romagnani S. (2000). T-cell subsets (Th1 versus Th2). Ann. Allergy Asthma Immunol..

[65] Han Q., Tong J., Sun Q., Teng X., Zhang H., Teng X. (2020). The involvement of miR-6615-5p/Smad7 axis and immune imbalance in ammonia-caused inflammatory injury via NF-κB pathway in broiler kidneys. Poult. Sci..

[66] Hu X., Chi Q., Wang D., Chi X., Teng X., Li S. (2018). Hydrogen sulfide inhalation-induced immune damage is involved in oxidative stress, inflammation, apoptosis and the Th1/Th2 imbalance in broiler bursa of Fabricius. Ecotox. Environ. Saf..

[67] Zhao F., Qu J., Wang W., Li S., Xu S. (2020). The imbalance of Th1/Th2 triggers an inflammatory response in chicken spleens after ammonia exposure. Poult. Sci..

[68] Liu L., Shen J., Zhao C., Wang X., Yao J., Gong Y., Yang X. (2015). Dietary Astragalus polysaccharide alleviated immunological stress in broilers exposed to lipopolysaccharide. Int. J. Biol. Macromol..

[69] Dong N., Li X., Xue C., Wang C., Xu X., Bi C., Shan A., Li D. (2019). Astragalus polysaccharides attenuated inflammation and balanced the gut microflora in mice challenged with Salmonella typhimurium. Int. Immunopharmacol..

[70] Farag M.R., Elhady W.M., Ahmed S.Y., Taha H.S., Alagawany M. (2019). Astragalus polysaccharides alleviate tilmicosin-induced toxicity in rats by inhibiting oxidative damage and modulating the expressions of HSP70, NF-kB and Nrf2/HO-1 pathway. Res. Vet. Sci..

[71] Lambrou G.I., Hatziagapiou K., Vlahopoulos S. (2020). Inflammation and tissue homeostasis: The NF-κB system in physiology and malignant progression. Mol. Biol. Rep..

[72] Hoffmann A., Baltimore D. (2006). Circuitry of nuclear factor κB signaling. Immunol. Rev..

[73] Zhang L., Xiao X., Arnold P.R., Li X.C. (2019). Transcriptional and epigenetic regulation of immune tolerance: Roles of the NF-κB family members. Cell Mol. Immunol..

[74] Chawla M., Roy P., Basak S. (2020). Role of the NF-κB system in context-specific tuning of the inflammatory gene response. Curr. Opin. Immunol..

[75] Surai P.F., Kochish I.I., Kidd M.T. (2021). Redox homeostasis in poultry: Regulatory roles of NF-κB. Antioxidants.

[76] Cheng P., Wang T., Li W., Muhammad I., Wang H., Sun X., Yang Y., Li J., Xiao T., Zhang X. (2017). Baicalin alleviates lipopolysaccharide-induced liver inflammation in chicken by suppressing TLR4-mediated NF-κB pathway. Front. Pharmacol..

[77] Tong Y., Yu C., Xie Z., Ziang X., Yang Z., Wang T. (2022). Trans-anethole ameliorates lipopolysaccharide-induced acute liver inflammation in broilers via inhibiting NF-κB signaling pathway. Poult. Sci..

[78] Zhen W., Shao Y., Wu Y. (2020). Dietary yeast β-glucan supplementation improves eggshell color and fertile eggs hatchability as well as enhances immune functions in breeder laying hens. Int. J. Biol. Macromol..

[79] AOAC (2012). Official methods of analysis of the Association of Official Analytical Chemists. https://www.aoac.org/.

[80] Ishida H. (1987). Quantitative surface FT-IR spectroscopic analysis of polymer. Rubber Chem. Technol..

[81] Saeed M., Abbas G., Alagawany M., Kamboh A.A., Abd El-Hack M.E., Khafaga A.F., Chao S. (2019). Heat stress management in poultry farms: A comprehensive overview. J. Therm. Biol..

[82] (2004). Chicken Feeding Standard.

[83] Gao X., Xiao Z.H., Liu M., Zhang N.Y., Khalil M.M., Gu C.Q., Qi D.S., Sun L.H. (2018). Dietary silymarin supplementation alleviates zearalenone-induced hepatotoxicity and reproductive toxicity in rats. J. Nutr..

[84] Luo J., Liu H., Wang J., Li L., Han C., Gan X., Li Y., Bai L., Mustafa A. (2018). Transcriptome reveals B lymphocyte apoptosis in duck embryonic bursa of Fabricius mediated by mitochondrial and Fas signaling pathways. Mol. Immunol..

[85] Livak K.J., Schmittgen T.D. (2001). Analysis of relative gene expression data using real-time quantitative PCR and the 2(-Delta Delta C(T)) Method. Methods.

